# Left ventricular to left arial volume ratio in the assessment of filling pressure in patients with dyspnoea and preserved ejection fraction

**DOI:** 10.3389/fcvm.2024.1357006

**Published:** 2024-02-09

**Authors:** Przemysław Palka, Roland Hilling-Smith, Rohan Swann, Sean Allwood, Alexander Moore, Chris Bian, Aleksandra Lange

**Affiliations:** ^1^ Queensland Cardiovascular Group, Brisbane, QLD, Australia; ^2^ Cardiac Catheterisation Laboratory, St Andrew's War Memorial Hospital, Brisbane, QLD, Australia

**Keywords:** dyspnoea, ejection fraction, left atrial pressure, filling pressure, echocardiography, left ventricle to left atrial volume ratio, diastasis

## Abstract

**Introduction:**

Assessing filling pressure (FP) remains a clinical challenge despite advancements in non-invasive imaging techniques. This study investigates the utility of echocardiographic left ventricular (LV) to left atrial (LA) volume ratio in estimating the resting FP in patients with dyspnoea and preserved ejection fraction (EF).

**Methods:**

This study is a prospective, single-centre analysis of 53 consecutive patients with dyspnoea (New York Heart Association grade 2 or 3) and LVEF of ≥50% (mean age 71 ± 10 years) who underwent cardiac catheterisation, including direct measurement of LA pressure at rest using retrograde technique. Echocardiographic data were obtained 1.5 ± 1.0 h after cardiac catheterisation. The patients were divided into two groups: Group 1 consisted of individuals with elevated FP, indicated by a mean LA pressure or mean pulmonary capillary wedge pressure of >12 mmHg, and Group 2 comprised of patients with normal FP. The LV and LA volumes were measured at three specific points: the minimum volume (LV_ES_, LA_min_), the volume during diastasis (LV_dias_, LA_dias_), and the maximum volume (LV_ED_, LA_max_). The corresponding LV/LA volume ratios were analysed: end-systole (LV_ES_/LA_max_), diastasis (LV_dias_/LA_dias_), and end-diastole (LV_ED_/LA_min_).

**Results:**

The patients in Group 1 exhibited lower LV/LA volume ratios compared with those in Group 2 (LV_ES_/LA_max_ 0.44 ± 0.12 vs. 0.60 ± 0.23, *P* = 0.0032; LV_dias_/LA_dias_ 1.13 ± 0.30 vs. 1.56 ± 0.49, *P* = 0.0007; LV_ED_/LA_min_ 2.71 ± 1.57 vs. 4.44 ± 1.70, *P* = 0.0004). The LV/LA volume ratios correlated inversely with an increased FP (LV_ES_/LA_max_, *r* = −0.40, *P* = 0.0033; LV_dias_/LA_dias_, *r* = −0.45, *P* = 0.0007; LV_ED_/LA_min_, *r* = −0.55, *P* < 0.0001). Among all the measurements, the LV_dias_/LA_dias_ ratio demonstrated the highest discriminatory power to distinguish patients with elevated FP from normal FP, with a cut-off value of ≤1.24 [area under the curve (AUC) = 0.822] for the entire group, encompassing both sinus rhythm and atrial fibrillation. For patients in sinus rhythm specifically, the cut-off value was ≤1.28 (AUC = 0.799), with *P* < 0.0001 for both. The LV_dias_/LA_dias_ index demonstrated non-inferiority to the E/e' ratio [ΔAUC = 0.159, confidence interval (CI) = −0.020–0.338; *P* = 0.0809], while surpassing the indices of LA reservoir function (ΔAUC = 0.249, CI = 0.044–0.454; *P* = 0.0176), LA reservoir strain (ΔAUC = 0.333, CI = 0.149–0.517; *P* = 0.0004), and LA_max_ index (ΔAUC = 0.224, CI = 0.043–0.406; *P* = 0.0152) in diagnosing patients with elevated FP.

**Conclusion:**

The study presents a straightforward and reproducible method for non-invasive estimation of FP using routine TTE in patients with dyspnoea and preserved EF. The LV_dias_/LA_dias_ index emerges as a promising indicator for identifying elevated FP, demonstrating comparable or even superior performance to established parameters.

## Introduction

Elevated filling pressure (FP) contributes to dyspnoea and is central to heart failure (HF) pathogenesis (1). Accurate FP approximation is crucial for therapy optimisation and prognosis determination (2, 3). Cardiac catheterisation, the gold standard, is invasive and impractical for serial monitoring in all patients (4). Over the past three decades, extensive research has focused on a non-invasive, rapid, or bedside screening tool for FP assessment. The evaluation of left atrial (LA) pressure or pulmonary capillary wedge pressure, as opposed to left ventricular (LV) end-diastolic pressure, provides a more accurate reflection of the elevated FP (5, 6). This differentiation is essential as it ensures a more precise understanding of FP, as LV end-diastolic pressure primarily conveys information regarding LV compliance and preload. The current guidelines from the American Society of Echocardiography and the European Association of Cardiovascular Imaging (ASE/EACVI) advocate for the evaluation of mean left atrial (LA) pressure. This assessment involves a composite score derived from selected indices acquired through Doppler blood flow, tissue Doppler imaging, and LA volume measurements (7, 8). Recent data underscore that in patients falling into the indeterminate category, the inclusion of LA strain measurements can further enhance diagnostic accuracy (9, 10). Patients presenting suboptimal diagnostic image quality, atrial fibrillation, conduction abnormalities, and mitral annulus calcification pose challenges within the non-invasive assessment paradigm (11). Thus, the overall efficacy of the non-invasive approach in routine clinical settings remains challenging (12, 13). In our recent work, we used computed tomography images to demonstrate that patients with abnormal diastolic function, as determined by ASE/EACVI criteria and/or documented elevated LV end-diastolic pressure, exhibit a decrease in the ratio of LV-to-LA volume measured during diastasis, specifically below 1.40 (14). Therefore, we sought to test whether, in patients with dyspnoea and preserved LV ejection fraction (EF), transthoracic echocardiogram (TTE)-derived measurements of LV/LA volume ratio taken at predefined phases of the cardiac cycle, including the period of diastasis, could effectively estimate FP. To validate our findings, the TTE results were compared with measurements obtained from right and left heart catheterisation, including direct retrograde LA pressure.

## Methods

### Selection criteria

Between January 2021 and February 2022, out of a consecutive group of 66 patients referred to the Queensland Cardiovascular Group in Brisbane, Australia, for cardiac catheterisation, 53 patients were enrolled and prospectively analysed. The study cohort exhibited a diverse range of underlying pathologies, including ischaemic and non-ischaemic conditions, hypertension, type II diabetes, with or without atrial fibrillation, and accompanying conduction abnormalities. The inclusion criteria consisted of the presence of dyspnoea (New York Heart Association grade II or III), preserved LVEF (≥50%), and no underlying valvular disease (grade >2/4). Patients with hypertrophic cardiomyopathy and/or cardiac amyloidosis were also excluded. A total of 12 patients were excluded from the original referral group for the following reasons: five patients had an LVEF of <50% on restudy evaluation, three patients required coronary artery interventions due to documented obstructive coronary artery disease, and four patients had non-cardiac issues.

To mitigate potential bias, the study was conducted in a consecutive group of patients who were referred for a haemodynamic assessment, and all data were collected prospectively. Exclusion was minimal, with only one patient omitted due to the inadequate quality of the two-dimensional TTE image. All other patients with satisfactory TTE image quality were included in the study. The time interval between cardiac catheterisation and TTE was minimised, ensuring a close temporal alignment. None of the enrolled patients exhibited clinical instability, and there were no alterations in the therapeutic regimen during the period between cardiac catheterisation and TTE. The TTE was conducted in the cardiac catheterisation laboratory within an average of 1.5 ± 1.0 h after the haemodynamic study.

The average age of the participants was 71 ± 10 years, and 49% of them were female. Out of the total 53 patients included in the study, 15 patients (28%) had a history of previous coronary artery revascularisation (either percutaneous coronary artery intervention and/or coronary artery bypass graft surgery), 38 patients (72%) had hypertension, and 12 patients (23%) had type II diabetes.

### Cardiac catheterisation

In all 53 patients, six French catheters were used for left heart catheterisation with the following access distribution: 48 patients had a right radial artery access, four patients had a right femoral artery access, and one patient had a left femoral artery access. Right heart catheterisation was performed in 43/53 patients. Six French catheters were used in 42 patients, whereas seven French catheters were used in one patient. In 38 cases, the right brachial vein was used as the access point, whereas the right femoral vein was used in four cases and the left femoral vein was used in one case. The direct measurements of LA pressure were obtained via the retrograde LV approach using a TIG catheter with side holes (15). All invasive haemodynamic measurements were acquired in a steady state during end-expiratory cycles*.* The offline measurements obtained from the LA pressure tracing include the mean LA pressure and the peaks observed during the V-wave, C-wave, and A-wave (patients in sinus rhythm). The measurements of LV end-diastolic pressure, mean right atrial pressure, pulmonary artery pressure, pulmonary artery wedge pressure, and pulmonary vascular resistance were obtained and calculated in a routine and standard manner.

### TTE

**Figure 1:**
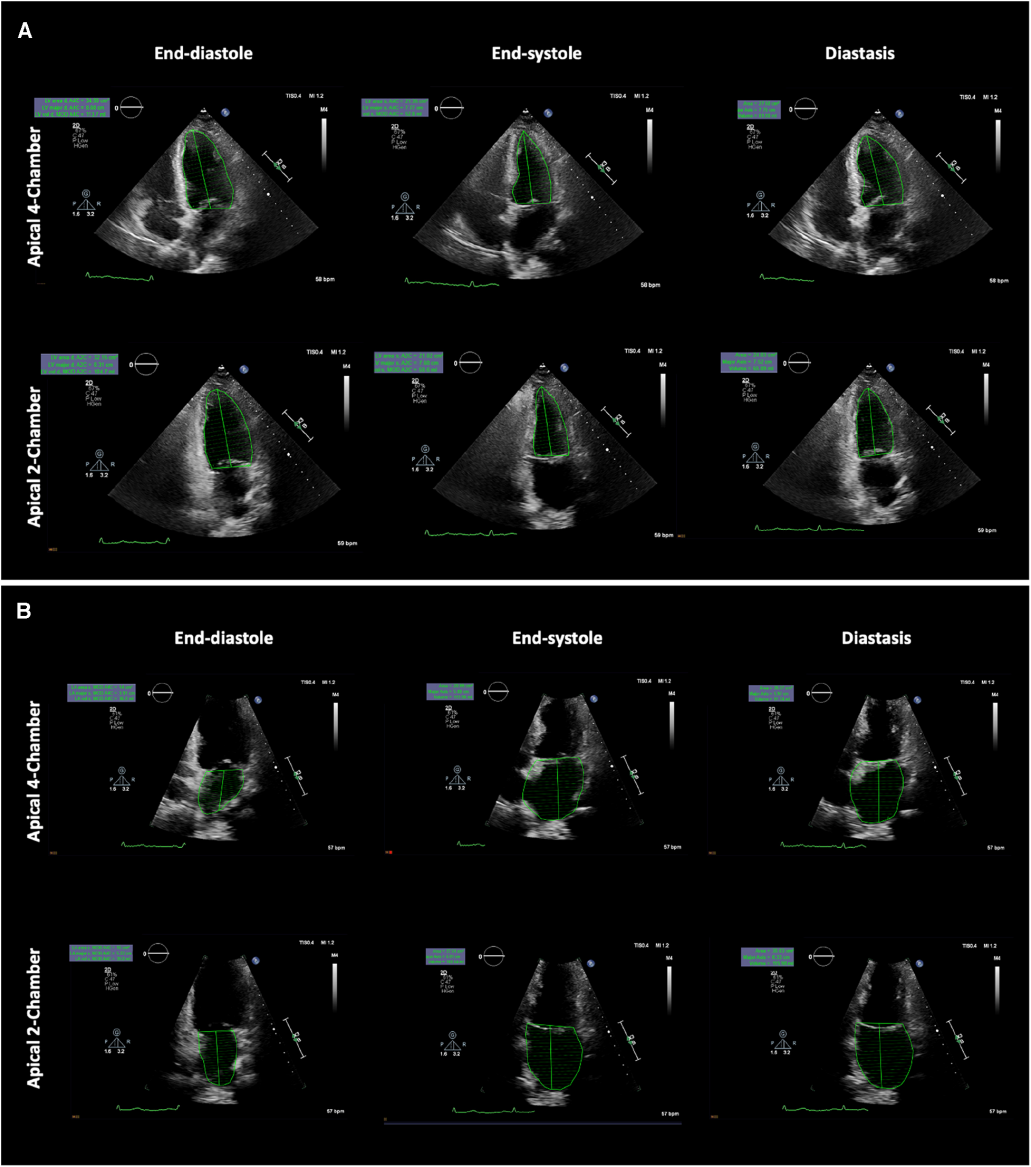
Example showing left ventricular and left atrial volume measurements at the specific phases of the cardiac cycle. (**A**) Left ventricle biplane Simpson's method (top panel apical four-chamber view, bottom panel apical two-chamber view) and (**B**) Left atrial biplane Simpson's method (top panel apical four-chamber view, bottom panel apical two-chamber view). LV and LA volumes were measured at the end-diastole (left panel), end-systole (middle panel), and in diastasis (right panel). LV, left ventricular; LA, left atrial.

**Figure 2:**
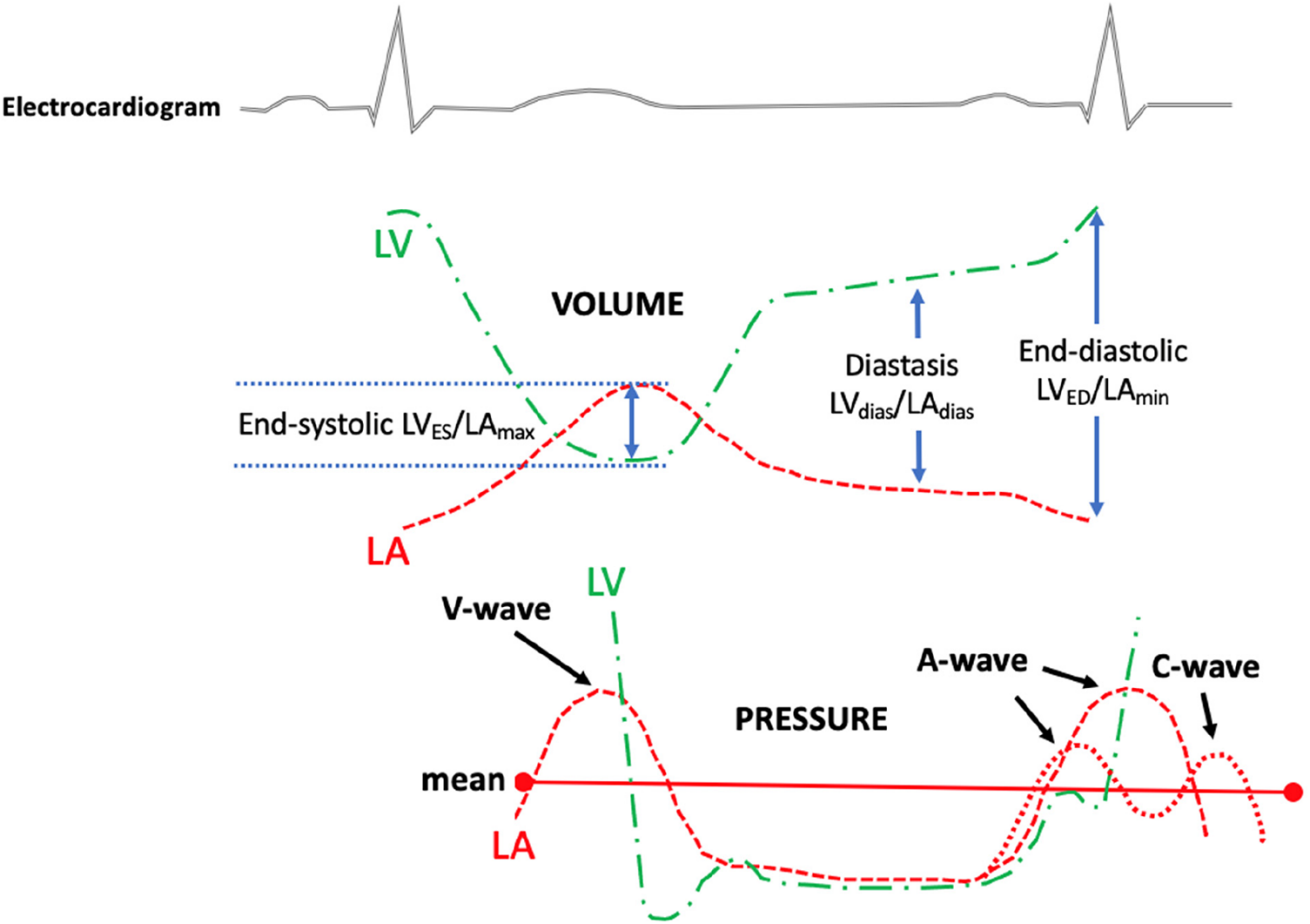
The diagram illustrates the measurement of the left ventricular to left atrial volume ratio (top panel) and the left atrial and left ventricular pressure (bottom panel). The left ventricular to left atrium volume ratio was measured from a routine transthoracic echocardiogram at certain phases of the cardiac cycle. The top dotted green line indicates LV volume changes during the cardiac cycle; the top red dotted line indicates LA volume changes during the cardiac cycle; the bottom green line indicates LV pressure changes during the cardiac cycle; and the bottom red dotted line indicates LA pressure changes during the cardiac cycle. LV, left ventricular/left ventricle; LA, left atrial; LV_ES_, left ventricular end-systolic volume; LV_ED_, left ventricular end-diastolic volume; LV_dias_, left ventricular diastasis volume; LA_max_, left atrial maximum volume; LA_min_, left atrial minimum volume; LA_dias_, left atrial diastasis volume.

The studies were acquired using commercially available equipment (Philips EPIQ CVx—Philips North America Corporation, Andover, Massachusetts, and Siemens SC2000—Siemens Medical Solutions, Mountain View, California). A standard imaging protocol, which included assessments of LV longitudinal strain and LA reservoir strain, was implemented for each patient. Apical views were meticulously optimised to prevent the foreshortening of both the LV and the LA. As a result, LV and LA volumes were measured in separate apical views. The LA volumes were measured using biplane Simpson’s method (from the four-chamber and the two-chamber views), excluding the LA appendage and the pulmonary veins (16). LA volumes were measured at the (i) maximum (LA_max_), (ii) diastasis (LA_dias_), and (iii) minimum (LA_min_) (17). Routine biplane Simpson's method was used to quantify LV volumes in (i) end-diastole (LV_ED_), (ii) end-systole (LV_ES_), and (iii) diastasis (LV_dias_), Figure 1. The diastasis period was determined through a visual assessment of the mitral valve motion and/or by considering the percentage of the cardiac cycle, as determined from Doppler mitral inflow velocities, and supported by a simultaneous electrocardiogram tracing. The measurements of LV_dias_ and LA_dias_ were consistently taken at 79% ± 6% (with a range of 65%–91%) of the cardiac cycle, irrespective of whether patients were in sinus rhythm or atrial fibrillation. All the measurements were averaged from three end-expiratory cardiac cycles in patients in sinus rhythm and from five end-expiratory cardiac cycles in patients with atrial fibrillation. As a result, three LV/LA ratios were calculated: end-systole (LV_ES_/LA_max_), diastasis (LV_dias_/LA_dias_), and end-diastole (LV_ED_/LA_min_), as depicted in Figure 2. To ensure quality and objectivity, all TTE measurements were conducted by very experienced sonographers, and echocardiographic data were independently and blindly analysed by RS, SA, and AM without access to the cardiac catheterisation results.

The study complies with the Declaration of Helsinki. The study was approved by the UnitingCare Human Research Ethics Committee (number 2021.01.339), and informed consent has been obtained from all patients.

## Statistical analysis

Continuous variables are expressed as mean ± standard deviation, and categorical variables are expressed as numbers and percentages. The sample power for testing the hypothesis that there will be a difference in the LV/LA volume ratio between the two groups (with a significance level of ∝ < 0.05) was calculated to be 0.934 for the potential 50 patients based on data from the first 12 patients. An unpaired *t*-test, chi-square, or Fisher exact test was used, when appropriate, for comparison in clinical, echocardiographic, and haemodynamic data. Linear regression and Pearson correlation were used to assess the relationship between echocardiographic variables and FP. For the assessment of predictive accuracy in predicting elevated FP, logistic regression analysis with power analysis was used to determine the area under the curve. Univariate linear regression with Pearson correlation coefficients was used to evaluate the relationship between FP and various TTE variables, including LV/LA ratios. A multivariate analysis of covariance (MANOVA) with the D'Agostino–Pearson test for normal distribution was employed to conduct a comprehensive linear regression comparison between LV_dias_/LA_dias_ and clinical variables, including FP. In addition, multivariate analysis of covariance was applied to examine the interdependence of TTE indices in assessing elevated FP. Receiver operating curve comparisons were performed by the DeLong et al. method (1988). Sensitivity and specificity were calculated in a standard manner. Intraobserver and interobserver variability in LV_dias_, LA_dias_, and the ratio of LV_dias_/LA_dias_ were assessed using the Bland and Altman method [mean difference ±2 standard deviation, 95% confidence interval (CI)]. All tests were two-sided, with a value of *P* < 0.05 considered significant. All analyses were performed by JMP 15 (SAS Institute, Cary, NC, USA) and MedCalc version 22.009 (Ostend, Belgium).

## Results

The patients were divided into two groups: Group 1 consisted of 29 (55%) patients with elevated FP (with a mean LA pressure or pulmonary capillary wedge pressure of >12 mmHg), and Group 2 comprised 24 (45%) patients with normal FP (with a mean LA pressure or pulmonary capillary wedge pressure of ≤12 mmHg). Out of the entire cohort, direct LA pressure was measured in 44 (83%) patients, and in the remaining nine (17%) patients, the mean pulmonary capillary wedge pressure was analysed.

Table 1 presents the clinical characteristics of the study patients. No significant differences were observed in basic clinical characteristics, including age (Group 1 73 ± 9 years vs. Group 2 70 ± 11 years, *P* = 0.2666), gender, history of underlying hypertension, and/or type II diabetes. However, the body mass index was higher in Group 1 (30 ± 5 kg/m^2^) compared with that of Group 2 (26 ± 4 kg/m^2^, *P* = 0.0057). The patients in Group 1 had a higher prevalence of stage 3 chronic kidney disease (52% vs. 17%, *P* = 0.006), elevated levels of N-terminal-prohormone of brain natriuretic peptide (902 ± 1,226 pg/ml vs. 294 ± 346 pg/ml; *P* = 0.0216), and a greater frequency of using loop and/or thiazides diuretics (41% vs. 17%; *P* = 0.0467). There were no significant differences observed in the use of other medications, but there was a tendency for a more frequent use of aldosterone antagonists and sodium-glucose transport protein 2-receptor inhibitors in Group 1 compared with Group 2. Furthermore, the patients in Group 1 exhibited lower levels of high-density lipids and tended to be more treated for obstructive sleep apnoea. The documented history of paroxysmal atrial fibrillation was similar in both groups (Group 1, 31% vs. Group 2, 25%; *P* = 0.2493), but all patients with chronic/persistent atrial fibrillation (7/53) were in Group 1 (*P* = 0.0058).

**Table 1:** Patients’ characteristics.

Parameters	Group 1 (Elevated filling pressure) *n* = 29	Group 2 (Normal filling pressure) *n* = 24	*P*-value
Age, years	73 ± 9	70 ± 11	0.2866
Gender, Female	15 (52)	11 (46)	0.6766
Body surface area, m^2^	1.96 ± 0.24	1.88 ± 0.15	0.1430
Body mass index, kg/m^2^	30 ± 5	26 ± 4	0.0057
Systolic blood pressure, mmHg	141 ± 30	130 ± 25	0.1586
Diastolic blood pressure, mmHg	69 ± 14	66 ± 12	0.4186
Heart rate, beats per minutes	68 ± 13	63 ± 11	0.1373
History of revascularisation^a^	8 (28)	7 (29)	0.8989
Previous permanent pacemaker implantation	4 (14)	4 (17)	0.7715
Hypertension	22 (76)	16 (67)	0.5467
Diabetes (on medications)	8 (28)	4 (17)	0.3398
Paroxysmal atrial fibrillation	9 (31)	6 (25)	0.2493
Smoking history (all ex-smokers)	4 (14)	4 (17)	0.7715
Hypercholesterolemia	25 (86)	22 (92)	0.6779
Chronic obstructive airway disease	4 (14)	3 (13)	0.8898
Treated obstructive sleep apnoea	10 (34)	3 (13)	0.0576
Documented (non-invasively) pulmonary hypertension^b^	14 (48)	9 (38)	0.4298
Plasma biochemistry			
Haemoglobin, g/L	129 ± 18	136 ± 15	0.1581
Estimated Glomerular Filtration Rate <60, ml/min	15 (52)	4 (17)	0.0066
N-terminal-prohormone of Brain Natriuretic Peptide, pg/ml [median, range]	902 ± 1,226 [430, 20–4,404]	294 ± 346 [222, 10–1,710]	0.0216
Iron deficiency^c^	15 (52)	12 (50)	0.7972
Total cholesterol, mmol/L	3.8 ± 0.8	4.0 ± 1.0	0.3637
Low-density lipids, mmol/L	1.8 ± 0.7	2.1 ± 1.0	0.1187
High-density lipids, mmol/L	1.1 ± 0.3	1.4 ± 0.4	0.0291
Electrocardiogram			
Atrial fibrillation	7 (24)	0	0.0058
Pacing rhythm	4 (14)	4 (17)	0.7715
PQ interval, msec	191 ± 50	180 ± 38	0.4130
PQ interval >200 msec	5 (17)	5 (21)	0.8764
QRS duration, msec	103 ± 24	109 ± 31	0.3876
QRS duration >120 msec	6 (21)	9 (38)	0.1763
Medications:			
Loop diuretics and/or thiazides	12 (41)	4 (17)	0.0467
Aldosterone antagonists	13 (45)	5 (21)	0.0664
Sodium-glucose transport protein 2-receptors inhibitors	3 (10)	0	0.0523
ß-blockers	19 (66)	16 (67)	0.9293
Angiotensin-converting enzyme inhibitors or angiotensin receptor blockers	19 (66)	16 (67)	0.9299
Calcium channel blockers	7 (24)	7 (29)	0.6798
Vasodilators (nitrates, ∝-blockers)	4 (14)	3 (13)	0.8898
Statins	21 (72)	21 (88)	0.1697
Anticoagulation	14(48)	6(25)	0.0787

LA, left atrial.

^a^ Percutaneous coronary intervention with stent(s) and/or coronary artery bypass surgery.

^b^ Either at rest or during exercise stress echocardiogram.

^c^ Ferritin <100 μg/L or 100−299 μg/L if the transferrin saturation is <20%.

## Haemodynamic data

The average mean LA pressure and mean pulmonary capillary pressure were higher in Group 1 (19 ± 5 mmHg and 17 ± 5 mmHg, respectively) compared with Group 2 (9 ± 2 mmHg and 11 ± 3 mmHg, respectively), *P* < 0.0001 for all. Also, the peak of C-, V-, and A-waves of LA pressure, LV end-diastolic pressure, and mean pulmonary artery pressure were higher in Group 1 compared with Group 2. Between Group 1 and Group 2, there were no differences in pulmonary vascular resistance or transpulmonary pressure gradient (Table 2).

**Table 2:** Haemodynamic and echocardiographic data.

Parameter	Group 1 Elevated filling pressure	Group 2 Normal filling pressure	*P*-value
Left heart catheterisation: (*n* = 44)			
Mean LA pressure, mmHg	19 ± 5	9 ± 2	<0.0001
peak V-wave	30 ± 7	13 ± 5	<0.0001
peak A-wave^a^	23 ± 5	15 ± 3	<0.0001
peak C-wave	20 ± 7	7 ± 4	<0.0001
LV end-diastolic pressure, mmHg	18 ± 5	14 ± 4	0.0045
Right heart catheterisation: (*n* = 43)			
Mean right atrial pressure, mmHg	9 ± 4	7 ± 3	0.0683
Mean pulmonary artery pressure, mmHg	26 ± 7	18 ± 4	<0.0001
Mean pulmonary capillary wedge pressure, mmHg	17 ± 5	11 ± 3	<0.0001
Pulmonary vascular resistance, Woods Unit	1.9 ± 1.2	2.1 ± 1.2	0.5795
Transpulmonary pressure gradient, mmHg	9 ± 6	7 ± 3	0.2368
Echocardiogram: (*n* = 53)			
M-mode and Two-dimensional measurements			
LV wall thickness (mean), cm	1.0 ± 0.2	1.0 ± 0.1	0.3927
LV dimension, cm	4.7 ± 0.8	4.7 ± 0.7	0.8719
LV mass, g	169 ± 53	164 ± 53	0.7082
LV mass index, g/m^2^	86 ± 23	87 ± 27	0.8396
LV hypertrophy^b^	6 (21)	5 (21)	0.9898
LV concentricity indices			
Relative wall thickness	0.46 ± 0.14	0.43 ± 0.08	0.3149
LV mass/LV_ED_	2.0 ± 0.5	1.8 ± 0.6	0.5143
LV_ED_ index/LV wall thickness (mean)	45 ± 13	52 ± 15	0.0610
LV_ED_, ml	87 ± 22	95 ± 28	0.2374
LV_ED_ index, ml/m^2^	44 ± 10	51 ± 15	0.0689
LV_ES_, ml	36 ± 12	38 ± 14	0.6342
LV_ES_ index, ml/m^2^	18 ± 6	20 ± 7	0.3483
LV_dias_, ml	74 ± 21	80 ± 24	0.3826
LV_dias_ index, ml/m^2^	38 ± 10	43 ± 13	0.1424
LV stroke volume, ml	51 ± 12	58 ± 16	0.1026
LV stroke volume index, ml/m^2^	26 ± 6	31 ± 8	0.0245
LV ejection fraction, %	61 ± 6	59 ± 7	0.2951
Median [range 50%–73%]	58 [50–71]	62 [50–73]	
LA_max_, ml	87 ± 34	66 ± 18	0.0075
LA_max_ index, ml/m^2^	44 ± 15	35 ± 10	0.0139
LA_dias_, ml	70 ± 25	53 ± 15	0.0024
LA_dias_ index, ml/m^2^	35 ± 11	28 ± 7	0.0060
LA_min_, ml	47 ± 33	24 ± 10	0.0013
LA_min_ index, ml/m^2^	24 ± 16	13 ± 6	0.0018
LV/LA volume ratio			
LV_ES_/LA_max_ (end-systolic)	0.44 ± 0.12	0.60 ± 0.23	0.0032
LV_dias_/LA_dias_ (diastasis)	1.13 ± 0.30	1.56 ± 0.49	0.0007
LV_ED_/LA_min_ (end-diastolic)	2.71 ± 1.57	4.44 ± 1.70	0.0004
LA function^c^			
LA reservoir function			
LA total emptying volume	40 ± 17	42 ± 13	0.6913
LA total emptying fraction	0.50 ± 0.21	0.63 ± 0.11	0.0041
LA conduit function			
LA passive emptying volume	17 ± 17	13 ± 11	0.3292
LA passive emptying fraction	0.18 ± 0.15	0.18 ± 0.15	0.9933
Conduit volume	11 ± 14	16 ± 16	0.2408
LA pump function^a^			
LA active emptying volume	23 ± 18	29 ± 9	0.1662
LA active emptying fraction	0.37 ± 0.28	0.55 ± 0.13	0.0052
LV and LA Strain			
LV Global longitudinal strain, %	18 ± 3	17 ± 4	0.4000
LA Reservoir strain, %	22 ± 12	18 ± 12	0.2520
Doppler assessment			
Peak mitral E-wave velocity, cm/s	83 ± 24	67 ± 21	0.0138
E-wave Deceleration time, ms	198 ± 69	232 ± 56	0.0503
Peak mitral A-wave velocity, cm/s^a^	77 ± 29	75 ± 31	0.8142
E/A ratio^a^	1.1 ± 0.4	1.0 ± 0.3	0.2113
E/A ratio >1.1^a^	8 (36)	6 (25)	0.4028
Isovolumic relaxation time, ms	96 ± 21	99 ± 26	0.6628
Peak septal e’ velocity, cm/s	5.8 ± 1.7	6.4 ± 2.1	0.2669
Peak lateral e’ velocity, cm/s	8.0 ± 2.4	8.3 ± 2.9	0.6981
E/e’ (average), ratio	14 ± 7	11 ± 5	0.0817
E/e’ >9	23 (79)	10 (42)	0.0045
E/e’ >14	8 (28)	4 (17)	0.3398
A-wave—atrial reversal duration, ms^a^	−13 ± 46	2 ± 33	0.2306
A-wave and atrial reversal duration > −30, ms^a^	8 (28)	5 (21)	0.2713
Estimated right ventricular systolic pressure, mmHg	36 ± 9	22 ± 9	0.2395
Estimated right ventricular systolic pressure >35 mmHg	12 (41)	7 (29)	0.5130
Tricuspid regurgitation velocity >2.8 m/s	4 (14)	4 (17)	0.6415
Peak tight ventricular free wall s’ velocity, cm/s	11 ± 3	11 ± 2	0.8590
Diastolic dysfunction grade^d^			
Normal	7 (24)	13 (54)	0.0240
Abnormal	8 (28)	5 (21)	0.5679
Indeterminate	14 (48)	6(25)	0.0787

LV, left ventricular; LA, left atrium; LV_ED_, left ventricular end-diastolic volume; LV_ES_, left ventricular end-systolic volume; LV_dias_, left ventricular diastasis volume; LA_max_, left atrial maximum volume; LA_dias_, left atrial diastasis volume; LA_min_, left atrial minimum volume.

^a^ Only in sinus rhythm.

^b^ LV mass index >95 for women and >115 for men, g/m^2^.

^c^ As per Blume et al. (17).

^d^ As per Nagueh et al. (7).

## TTE data

### The left ventricle

When comparing all recorded LV measurements, it was observed that only the LV stroke volume index exhibited a lower value in Group 1 compared with Group 2 (26 ± 6 ml/m^2^ vs. 31 ± 8 ml/m^2^; *P* = 0.0245). There were no differences found between Groups 1 and 2 in LV wall thickness, LV mass, LV mass index, LV hypertrophy, and LV concentricity indices (Table 2).

### The left atrium

In Group 1, all measured LA volumes were higher compared with Group 2 (LA_max_ 87 ± 34 ml vs. 66 ± 18 ml, *P* = 0.0075; LA_dias_ 70 ± 25 ml vs. 53 ± 15 ml, *P* = 0.0024; LA_min_ 47 ± 33 ml vs. 24 ± 10 ml, *P* = 0.0013; respectively). A similar pattern was noted when measured LA volumes were indexed by body surface area (LA_max_ index 44 ± 15 ml/m^2^ vs. 35 ± 10 ml/m^2^, *P* = 0.0139; LA_dias_ index 35 ± 11 ml/m^2^ vs. 28 ± 7 ml/m^2^, *P* = 0.0060; LA_min_ index 24 ± 16 ml/m^2^ vs. 13 ± 6 ml/m^2^, *P* = 0.0018; respectively). Among LA function measurements, it was observed that only LA total emptying fraction (LA reservoir function) and LA active emptying fraction (LA pump function) were lower in Group 1 compared with Group 2 (0.50 ± 0.21 vs. 0.63 ± 0.11, *P* = 0.0041; and 0.37 ± 0.28 vs. 0.55 ± 0.13, *P* = 0.0052; respectively).

### Strain measurement

Both LV global longitudinal strain and LA reservoir strain were similar in Group 1 and Group 2 (18% ± 3% vs. 17% ± 4%, *P* = 0.4000; and 22 ± 12 vs. 18 ± 12, *P* = 0.2520; respectively).

### Doppler measurements

Group 1 exhibited a higher mitral inflow peak E-wave velocity compared with Group 2 (83 ± 24 cm/s vs. 67 ± 21 cm/s, *P* = 0.0138) and a higher proportion of patients with an E/e' ratio of >9 (79% vs. 42%, *P* = 0.0045). The percentage of patients with abnormal diastolic function (ASE/EACVI 2016 guidance) (7) did not differ between Group 1 and Group 2 (28% vs. 21%, *P* = 0.5679), but Group 2 had a higher proportion of patients with normal diastolic function compared with Group 1 (54% vs. 24%, *P* = 0.0240).

### The ratio of left ventricle to left atrium volume

The LV/LA volume ratios were found to be lower in Group 1 for all three measurements.

1. The ratio of minimum LV to maximum LA volumes (LV_ES_/LA_max_) was 0.44 ± 0.12 in Group 1 and 0.60 ± 0.23 in Group 2 (*P* = 0.0032).
2. The ratio taken in LV diastasis (LV_dias_/LA_dias)_ was 1.13 ± 0.30 in Group 1 and 1.56 ± 0.49 in Group 2 (*P* = 0.0007).
3. The ratio of maximum LV to minimum LA volumes (LV_ED_/LA_min_) was 2.71 ± 1.57 in Group 1 and 4.44 ± 1.70 in Group 2 (*P* = 0.0004).

**Figure 3:**
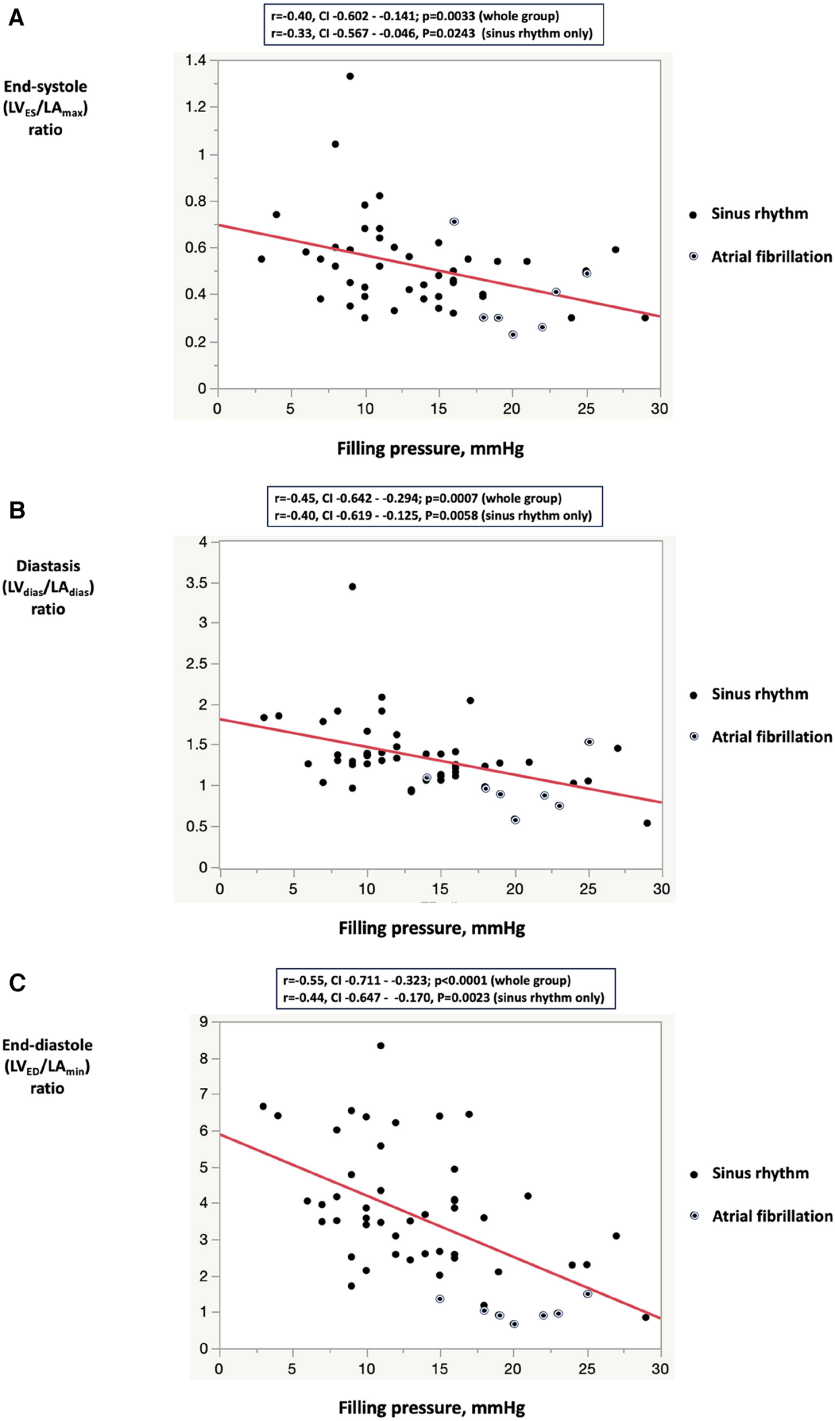
Correlation between filling pressure and the left ventricular to left atrial volume ratio indices: (**A**) left ventricular end-systolic volume to left arial maximum volume ratio; (**B**) left ventricular diastasis volume to left atrial diastasis volume ratio; (**C**) left ventricular end-diastolic volume to left atrial minimum volume ratio. In the sinus rhythm subgroup analysis, all three LV/LA volume indexes were lower in Group 1 compared with Group 2 (LV_ES_/LA_max_ 0.46 ± 0.10 vs. 0.60 ± 0.23, *P* = 0.0087; LV_dias_/LA_dias_ 1.19 ± 0.28 vs. 1.56 ± 0.49, *P* = 0.0029, LV_ED_/LA_min_ 3.24 ± 1.43 vs. 4.44 ± 1.70, *P* = 0.0123). LV_ES_, left ventricular end-systolic volume; LV_ED_, left ventricular end-diastolic volume; LV_dias_, left ventricular diastasis volume; LA_max_, left atrial maximum volume, LA_min_, left atrial minimum volume; LA_dias_, left atrial diastasis volume.

Figure 3 illustrates an inverse correlation between the reduction in all three LV/LA ratio indices and the values of FP. The correlation between LV/LA ratio indices and FP remained consistent regardless of the underlying rhythm, whether it was sinus rhythm or atrial fibrillation. This independence strengthens the reliability of the LV/LA ratio indices as markers for FP, regardless of the patient's rhythm status.

The analysis of all the TTE parameters revealed that each of the three LV/LA volume ratio indices—LV_ES_/LA_max_, LV_dias_/LA_dias_, and LV_ED_/LA_min—_exhibited high discriminatory ability to diagnose patients with elevated FP. With cut-off values of ≤1.24 for LV_dias_/LA_dias_, ≤2.66 for LV_ED_/LA_min_, and ≤0.50 for LV_ES_/LA_max_, they demonstrated overall accuracies of 0.81, 0.73, and 0.73, respectively (Table 3A). The observed decrease in the LV/LA volume ratio in patients with elevated FP persists in the subgroup analysis limited to patients in sinus rhythm (Table 3B). Specifically, the LV_dias_/LA_dias_ ratio maintains its prominence with the highest area under the curve (AUC) of 0.822 (for all patients) and of 0.799 (for patients with sinus rhythm), effectively distinguishing between those with elevated and normal FP. For patients in sinus rhythm, the assessment of LA volume measurements alone, including LA_max_, LA_dias_, and LA_min_ (both absolute and index), as well as LA reservoir function and LA reservoir strain, demonstrated limited accuracy in distinguishing between individuals with elevated and normal FP when compared with the LV/LA volume ratio assessment. In the subgroup analysis of patients in sinus rhythm, the LV_dias_/LA_dias_ measurement with a cut-off value of ≤1.28 emerged as the most accurate TTE parameter in distinguishing patients with elevated from normal FP (*P* < 0.0001).

**Table 3:** Power analysis of echocardiographic parameters to diagnose elevated filling pressure.

Parameters	Cut-off value	Sensitivity %	Specificity %	Accuracy %	AUC	95% confidence interval	*Z* statistics	*P*-value
(**A**) The whole group (patients in sinus rhythm and atrial fibrillation)								
LV_dias_/LA_dias_ (Diastasis), ratio	≤1.24	69	92	81	0.822	0.692–0.913	5.435	<0.0001
LV_ED_/LA_min_ (End-diastolic), ratio	≤2.66	62	83	73	0.768	0.632–0.873	4.153	<0.0001
LV_ES_/LA_max_ (End-systolic), ratio	≤0.50	76	71	73	0.751	0.613–0.859	3.583	0.0003
LA_min_, ml	>32	52	88	75	0.717	0.576–0.832	3.095	0.0020
LA_min_ index, ml/m^2^	>23	41	96	69	0.694	0.552–0.813	2.680	0.0074
LA_dias_, ml	>66	45	92	69	0.702	0.561–0.820	2.807	0.0050
LA_dias_ index, ml/m^2^	>26	86	42	64	0.680	0.537–0.801	2.787	0.0148
LA_max_, ml	>87	35	96	65	0.671	0.528–0.794	2.298	0.0215
LA_max_ index, ml/m^2^	>52	31	100	65	0.652	0.508–0.777	1.988	0.0468
E/e’ (average), ratio	>9	79	58	68	0.693	0.551–0.813	2.480	0.0131
LV stroke volume index, ml/m^2^	<33	97	46	71	0.713	0.572–0.829	2.797	0.0052
LV_ED_ index/mean LV wall thickness, ratio	≤49	72	67	69	0.672	0.530–0.795	2.204	0.0275
LA reservoir function^a^	≤0.39	35	100	68	0.695	0.554–0.814	2.699	0.0070
LA reservoir strain, %	≤10	36	86	61	0.600	0.447–0.740	1.188	0.2349
(**B**) Patients in sinus rhythm only								
LV_dias_/LA_dias_ (Diastasis), ratio	≤1.28	77	79	78	0.799	0.652–0.903	4.344	<0.0001
LV_ED_/LA_min_ (End-diastolic), ratio	≤3.09	55	79	67	0.694	0.541–0.821	2.464	0.0138
LV_ES_/LA_max_ (End-systolic), ratio	≤0.56	91	54	73	0.726	0.575–0.847	2.913	0.0036
LA_min_, ml	>32	36	88	62	0.627	0.472–0.765	1.516	0.1294
LA_min_ index, ml/m^2^	>10	77	46	62	0.597	0.442–0.739	1.130	0.2583
LA_dias_, ml	>65	41	88	65	0.649	0.494–0.783	1.790	0.0735
LA_dias_ index, ml/m^2^	>25	91	33	62	0.625	0.470–0.763	1.498	0.1342
LA_max_, ml	>60	77	46	61	0.601	0.446–0.743	1.186	0.2356
LA_max_ index, ml/m^2^	>52	18	100	59	0.575	0.420–0.719	0.864	0.3875
E/e’ (average), ratio	>7	91	42	67	0.667	0.512–0.799	2.016	0.0438
LV stroke volume index, ml/m^2^	≤33	95	46	71	0.672	0.518–0.803	2.094	0.0362
LV_ED_ index/mean LV wall thickness, ratio	≤49	73	67	70	0.652	0.497–0.786	1.793	0.0730
LA reservoir function^a^	≤0.63	59	63	61	0.598	0.444–0.740	1.151	0.2499
LA reservoir strain, %	≤24	75	36	56	0.526	0.366–0.682	0.287	0.7749

LA, left atrial; LV, left ventricle; LV_ES_, left ventricular end-systolic volume; LV_ED_, left ventricular end-diastolic volume; LV_dias_, left ventricular diastasis volume; LA_max_, left atrial maximum volume; LA_min_, left atrial minimum volume; LA_dias_, left atrial diastasis volume; E/e’, ratio of early diastolic mitral inflow to mitral annular tissue velocities; AUC, area under the curve.

^a^ Formula: (LA_max_–LA_min_)/LA_max_ (17).

### Multivariate analysis

The multivariate regression analysis revealed a strong association between the reduction in LV_dias_/LA_dias_ ratio and increased FP, with a weak correlation to patient age (Table 4). The decrease in the LV_dias_/LA_dias_ ratio was found to be independent of atrial fibrillation and other comorbidities, including obesity, underlying hypertension, type II diabetes, or obstructive sleep apnoea. Among all the TTE measurements studied, only the LV_dias_/LA_dias_ ratio demonstrated a significant correlation with the presence of elevated FP compared with other indices such as E/e', LA reservoir function, LA reservoir strain, LA_max_ index, and LA_min_ (correlation coefficient for multivariate comparison −0.528, standard error 0.181, *P* = 0.0058; with constant 1.496, coefficient of determination *R*^2^ 0.332, and residual standard deviation 0.442).

**Table 4:** Univariate and multivariate linear regression comparison between the echocardiographic left ventricular diastasis volume to left atrial diastasis volume ratio (LV_dias_/LA_dias_) and clinical variables.

Clinical variables	Univariate			Multivariate		
	*r*	Coefficient of regression	*P*-value	*β*	Standard Error	*P*-value
Filling pressure	−0.450	−0.034	0.0007	−0.030	0.010	0.0030
Age	−0.329	−7.124	0.0161	−0.011	0.006	0.0692
Atrial fibrillation	−0.321	−0.244	0.0192	–	–	–
Hypertension	−0.226	−0.228	0.1041	–	–	–
Body mass index	−0.218	−2.364	0.1177	–	–	–
N-terminal-prohormone of brain natriuretic peptide	−0.205	−441.043	0.1533	–	–	–
Diabetes	0.018	0.017	0.9007	–	–	–
Gender, female	−0.164	−0.184	0.2411	–	–	–
History of revascularisation	−0.012	−0.012	0.9303			

*r*, correlation coefficient for univariate comparison; *β*, correlation coefficient for multivariate comparison with constant 2.520, coefficient of determination (*R*^2^) 0.254, multiple correlation coefficient 0.504 and residual standard deviation 0.396.

**Figure 4:**
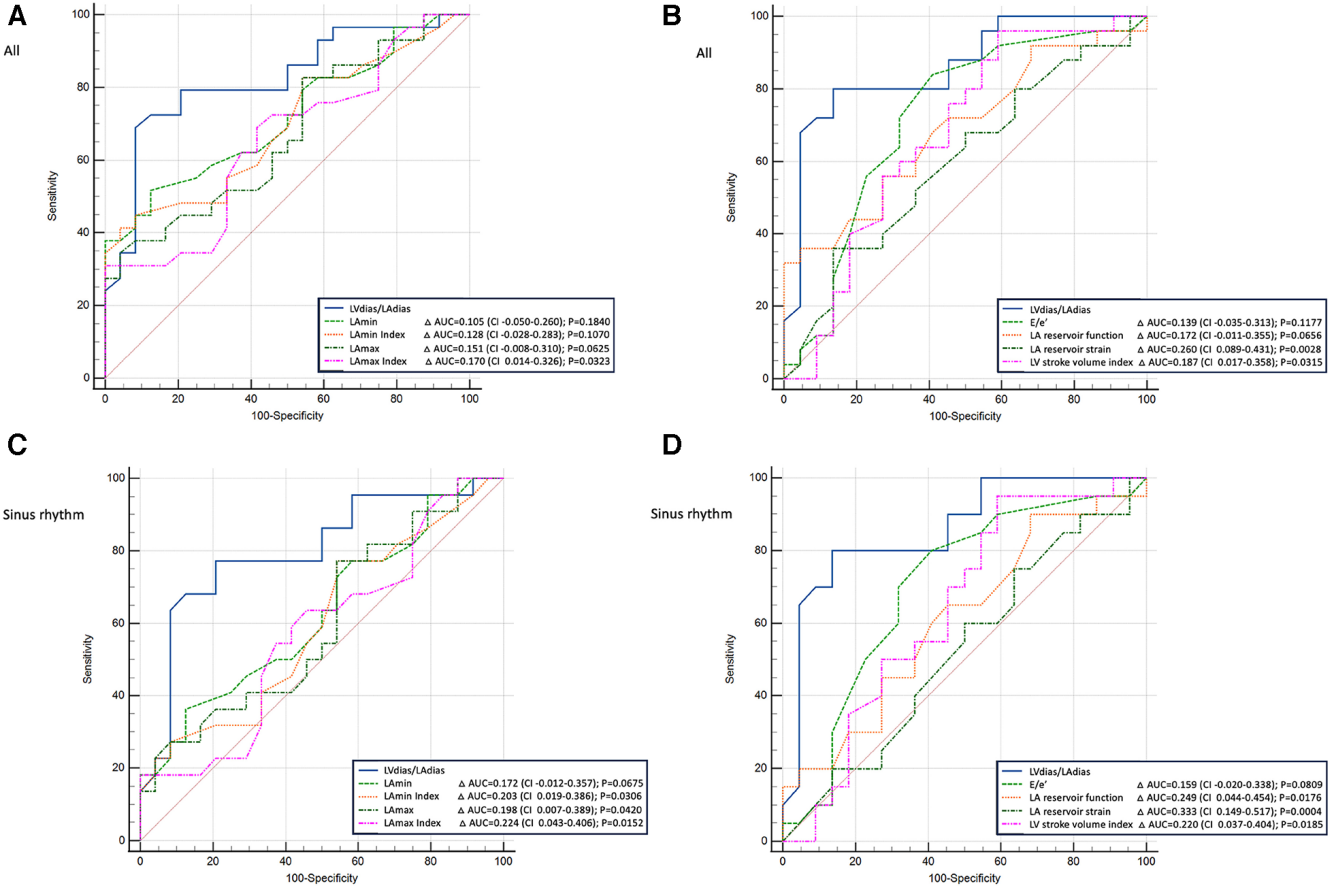
Comparison of power analysis of the receiver operating characteristic curves to diagnose patients with elevated filling pressure. (**A,B**) The whole group (patients with sinus rhythm or atrial fibrillation); (**C,D**) patients with sinus rhythm only. (**A,C**) The left ventricular diastasis volume to left atrial diastasis volume ratio compared with the left atrial maximum and minimum volumes (absolute values and index for body surface area). (**B,D**) The left ventricular diastasis volume to left atrial diastasis volume ratio compared with early diastolic mitral inflow to mitral annulus velocity ratio, left atrial reservoir function, left atrial reservoir strain, and left ventricle stroke volume index. LV_dias_/LA_dias_, left ventricular diastasis volume to left atrial diastasis volume ratio; E/e’, early diastolic mitral inflow to mitral annular tissue velocities ratio; LA_min_, left atrial minimal volume; LA_max_, left atrial maximum volume; CI, confidence interval; AUC, area under the curve.

The comparison of logistic regression analysis curves demonstrated that the LV_dias_/LA_dias_ ratio was non-inferior to the E/e' ratio in diagnosing patients with elevated FP (for all: ΔAUC = 0.139, CI = −0.014–0.313, *P* = 0.1177; for sinus rhythm: ΔAUC = 0.159, CI = −0.020–0.338, *P* = 0.0809, Figure 4). Particularly in patients in sinus rhythm, the LV_dias_/LA_dias_ ratio measurements outperformed the other studied TTE parameters, including LA_max_ index (ΔAUC = 0.224, CI = 0.043–0.406, *P* = 0.0152), LA reservoir function (ΔAUC = 0.249, CI = 0.044–0.454, *P* = 0.0176), LA reservoir strain (ΔAUC = 0.333, CI = 0.149–0.517, *P* = 0.0004), and LV stroke volume index (ΔAUC = 0.220, CI = 0.037–0.404, *P* = 0.0185).

## Interobserver and intraobserver agreement

The agreement between LV_dias_ and LA_dias_ measurements was evaluated in a subgroup of 15 randomly chosen patients. This assessment aimed to determine the consistency or similarity between these two measurements. The mean difference between two independent observers (interobserver variability) was 0 ± 10 ml for LV_dias_, −1 ± 10 ml for LA_dias_, and 0.03 ± 0.32 for LV_dias_/LA_dias_. The mean difference between two measurements performed by the same operator (intraobserver variability) was 0 ± 13 ml for LV_dias_, −1 ± 10 ml for LA_dias_, and 0.04 ± 0.32 for LV_dias_/LA_dias_.

## Discussion

This prospective study involved a consecutive group of patients with persistent symptoms despite optimal medical therapy who subsequently underwent cardiac catheterisation. Our findings suggest that in individuals experiencing shortness of breath, lacking significant valve disease, and maintaining preserved LVEF, a non-invasive estimation of FP is possible using the LV/LA volume ratio obtained from standard two-dimensional TTE images.

Patients with elevated FP, defined by a mean LA pressure and/or pulmonary capillary wedge pressure higher than 12 mmHg, also displayed elevated LV end-diastolic pressure but not pulmonary vascular resistance or transpulmonary gradient (3–6). Consistent with existing literature, patients with elevated FP were characterised by a higher body mass index, elevated natriuretic peptide levels, a higher prevalence of chronic kidney disease, and a more frequent occurrence of persistent/chronic atrial fibrillation (18, 19).

Among all LV measurements, only the indexed LV stroke volume was shown to be reduced in patients with elevated FP, supporting the previously observed inverse relationship between LA pressure and LV stroke volume (20). The presence of increased LV wall thickness as well as concentric LV remodelling did not provide informative distinctions between patients with elevated and normal FP. These findings are also supported by previous studies, demonstrating that although LV hypertrophy is often present in patients with elevated FP, it is a non-specific finding (21).

Historically, LA_max_ measurement has been documented as a powerful predictor of survival following myocardial infarction (22). Recent research has linked an increase in LA_min_ to a poor prognosis in HF with preserved EF (23). We have observed that all measured LA volumes (LA_max_, LA_dias_, LA_min_) were higher in patients with elevated FP. Differences were observed in LA reservoir function and LA pump function among the investigated LA function parameters, consistent with the findings reported by others (24). Notably, LA reservoir strain showed similar values in patients with normal and elevated FP, aligning with previous research indicating that LA reservoir strain is determined by LV strain (25) and has limited value in assessing FP in patients with preserved EF compared with those with reduced EF (10). The increase in LA volume throughout the cardiac cycle, measured at LA_max_, LA_dias_, and LA_min_, and the observed impairment in LA reservoir function and strain observed in our study confirmed that the presence of atrial fibrillation contributes to LA remodelling.

Our data confirmed a well-documented inverse relationship between E/e' and FP (19, 26), but the combination of the scores recommended by ASE/EACVI provided limited assistance in distinguishing patients with elevated from normal FP (7). However, we did not evaluate the potential pathological increase in FP during exercise, which could reveal underlying diastolic dysfunction in some patients with normal resting FP (27).

**Figure 5:**
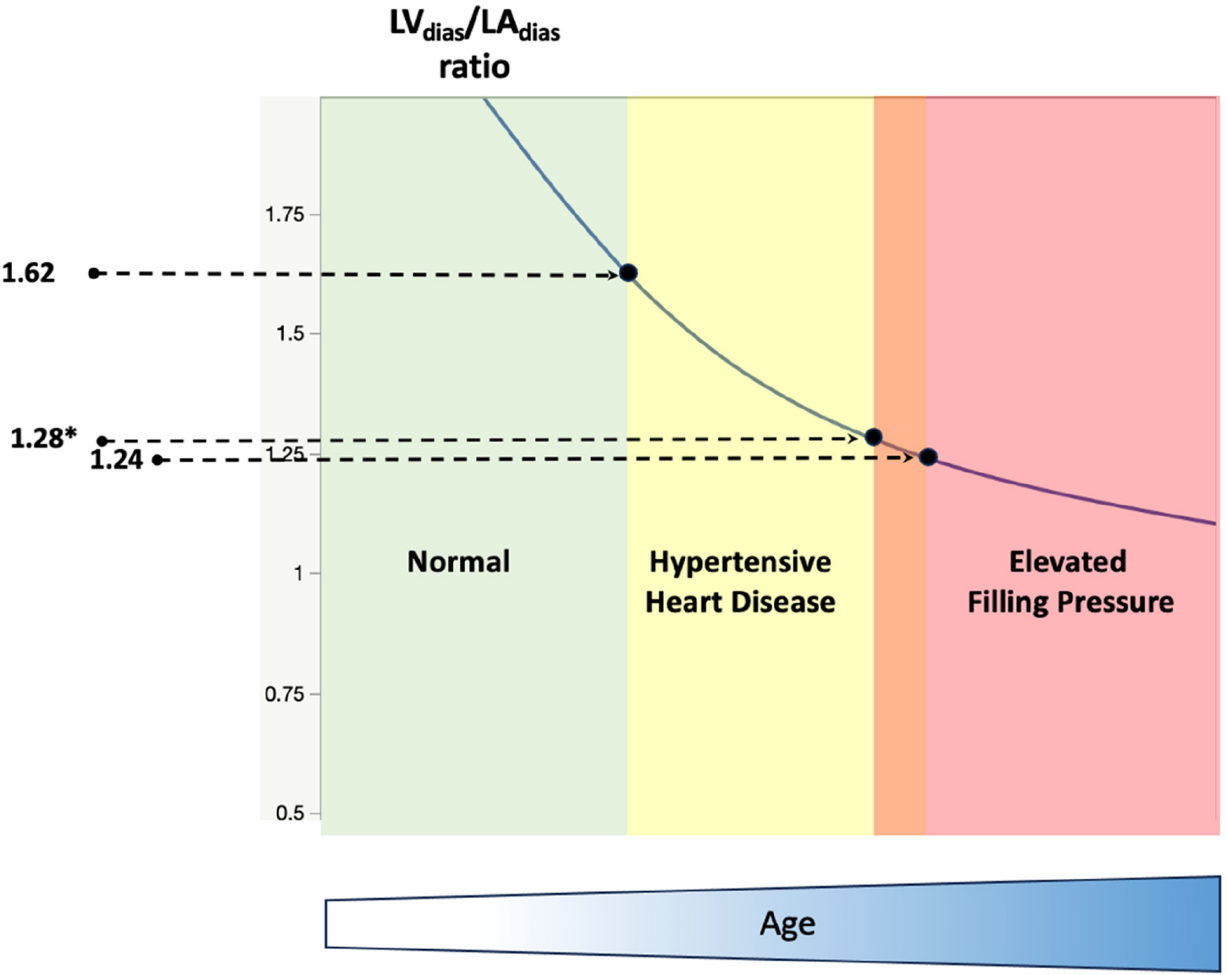
A schematic diagram illustrates the progressive reduction of the diastasis left ventricle to left atrial volume ratio in normal (left, green colour), hypertensive heart disease (middle, yellow colour), and patients with dyspnoea and elevated resting filling pressure (right, red colour). LV_dias_/LA_dias_, left ventricle diastasis volume to left atrial diastasis volume ratio. The cut-off value of LV_dias_/LA_dias_ to diagnose hypertensive heart disease is ≤1.62 (27), and the cut-off value to diagnose patients with elevated filling pressure is ≤1.24 (*≤1.28 for patients in sinus rhythm only). Note that the reduction in LV_dias_/LA_dias_ is also correlated with patients’ age (bottom, blue colour).

The study showed a decrease in all three measured LV/LA volume ratios in patients with elevated FP. Among the TTE indices studied, the LV_dias_/LA_dias_ ratio, with a cut-off value of ≤1.24 (for all patients, including those in atrial fibrillation) and ≤1.28 (for patients in sinus rhythm only) demonstrated the highest discriminatory ability for indicating elevated FP. This reduction in these LV/LA ratios correlated with the rate of increase in FP, as well as patients' age, as partially indicated in our previous work in patients with hypertension (28). A progressive reduction in the LV_dias_/LA_dias_ ratio was observed in hypertensive heart disease and in patients with elevated resting FP, both in sinus rhythm and/or atrial fibrillation. This finding supports the potential clinical relevance of this ratio, with cut-off values of ≤1.62, ≤1.28, and ≤1.24, respectively, as illustrated in Figure 5.

The LV_dias_/LA_dias_ ratio demonstrated superior performance compared with other TTE parameters, including LA_max_ index, LA reservoir function, LA reservoir strain, and LV stroke volume index. These findings underscore the potential utility of the LV_dias_/LA_dias_ ratio in assessing elevated FP. The LV/LA volume ratios demonstrated ease of measurements and good reproducibility, offering an attractive alternative to the current complex assessment of FP, which often requires combined information from different imaging modalities. An additional benefit is its consistent accuracy, unaffected by the underlying rhythm, whether it in is sinus rhythm or atrial fibrillation.

The reduced LV/LA ratio can be explained by the impaired net atrio-ventricular compliance due to chronic LA myopathic remodelling, with the LA wall becoming thinner and more compliant compared with a thicker and less compliant LV wall resulting from chronically elevated preload (29). The concept of the LV-to-LA volume ratio has been explored previously. Spevack et al. (30) found that in patients with hypertension or diabetes, the ratio of LA to LV diameter can serve as a marker of the diastolic LV pressure–volume relationship. Their findings align with the findings of our present study exploring LV/LA volume ratios as potential indicators of elevated FP. Recent studies in cardiac magnetic resonance imaging, conducted by Pezel et al. (31), demonstrated that the LA to LV volume ratio (LA_min_ to LV_ED_) could offer incremental prognostic value for predicting HF events beyond traditional risk factors. In addition, Garg et al. (32) suggested that elevated LV FP can be estimated from both LV mass and LA volume. Our study demonstrated a weak correlation between FP and either LV mass or LA_max_ acquired by TTE. It is important to note that we did not analyse any other cardiac imaging techniques, including cardiac magnetic imaging.

## Limitations

While our study involved a relatively small, consecutive group of patients, the inclusion of a small group of patients with chronic/persistent atrial fibrillation was intentional to address the diagnostic challenges posed by intrinsic atrial dysfunction, a key factor in HF (33, 34). We focused on patients with an LVEF cut-off value of ≥50%, and the potential role of LV/LA volume ratio indices in patients with reduced LVEF and/or valvular disease remains unknown. In addition, our cardiac catheterisation data were performed at rest, and potential exercise-related changes between FP and LV/LA volume ratio are unknown. The potential superiority of the proposed volumetric two-dimensional measurements, such as the LV_dias_/LA_dias_ ratio, over other echocardiographic measurements, particularly LA strain, may be attributed to the inclusion of all examined patients without excluding them based on TTE image quality. Both LV and LA chamber volumes were manually traced by experienced sonographers, in contrast to the semiautomatic nature of obtaining LV or LA strain. This may explain the relatively lower accuracy of the LA strain, particularly in detecting elevated FP, as it was not limited to selected patients with good TTE image quality. Nevertheless, our study results indicate that the assessment of FP volume using the LV_dias_/LA_dias_ ratio may be performed in essentially all patients, thereby providing a broader range of routine clinical applications.

Our study was performed on clinically stable patients. It is unknown whether our results could be incorporated in acute settings or in populations with significant valvular disease. Due to the fact that this was a single-centre study, further validation through a multicentre study should be considered.

### Future research pathways and potential clinical applications

Further research should explore the establishment of normal values for LV_dias_/LA_dias_ in various age subgroups, given that the present study focused on patients with a mean age of 71 years. Investigating the applicability of the LV_dias_/LA_dias_ ratio across a broader spectrum of diseases is essential. For instance, assessing early changes in specific age groups, such as patients with diabetes or hypertension, before the onset of HF symptoms (stage A or B) could provide valuable insights. Furthermore, there is a need to investigate whether the LV_dias_/LA_dias_ ratio can serve as a diastolic function parameter in developing machine-augmented echocardiography for diagnosing diastolic dysfunction and managing HF patients. This area requires additional study and may contribute to advancements in the diagnosis and management of HF (35, 36).

### Clinical relevance

Our study highlights the LV_dias_/LA_dias_ ratio as a straightforward volumetric assessment for evaluating FP in patients experiencing dyspnoea, particularly when a routine TTE indicates preserved LVEF (≥50%). This ratio is notable as a powerful and singular test that could significantly guide the management of HF patients. Monitoring changes in the LV_dias_/LA_dias_ ratio may potentially allow not only the assessment of disease progression but, more importantly, facilitate the monitoring of treatment efficacy. Presently, there is no single TTE parameter that serves as a definitive tool for both the diagnosis and monitoring of the effects of medications or procedures on FP. Hence, the LV_dias_/LA_dias_ ratio could serve as a dual-purpose diagnostic and management tool in both clinical practice and future research trials.

## Summary

This study suggests that in patients with dyspnoea and preserved LV ejection fraction, estimating FP is possible using the LV/LA volume ratio derived from routine TTE. The LV_dias_/LA_dias_ ratio demonstrated the highest discriminatory ability to differentiate between patients with elevated FP and normal FP. The proposed measures of the LV/LA volume ratio, which can be easily obtained and replicated using routine TTE images, could provide an alternative approach to the current complex assessment of FP.

## Data Availability

The raw data supporting the conclusions of this article will be made available by the authors, without undue reservation.
